# Efficacy evaluation of Lattice Carbon Dioxide Laser Therapy in the treatment of postmenopausal patients with mild to moderate stress urinary incontinence

**DOI:** 10.12669/pjms.37.7.4077

**Published:** 2021

**Authors:** Ya-ru Wu, Dan Shen, Yan-qiao Zhang, Zhen-yu Cui, Wen-zeng Yang

**Affiliations:** 1 Ya-ru Wu, Department of Urology, Affiliated Hospital of Hebei University, Baoding, Hebei, 071030, P. R. China; 2 Dan Shen, Department of Urology, Affiliated Hospital of Hebei University, Baoding, Hebei, 071030, P. R. China; 3 Yan-qiao Zhang, Department of Urology, Affiliated Hospital of Hebei University, Baoding, Hebei, 071030, P. R. China; 4 Zhen-yu Cui, Department of Urology, Affiliated Hospital of Hebei University, Baoding, Hebei, 071030, P. R. China; 5 Wen-zeng Yang Department of Urology, Affiliated Hospital of Hebei University, Baoding, Hebei, 071030, P. R. China

**Keywords:** Stress urinary incontinence, Lattice carbon dioxide laser, Menopause

## Abstract

**Objectives:**

To investigate the efficacy and postoperative complications of lattice carbon dioxide laser in the treatment of postmenopausal patients with mild to moderate stress urinary incontinence.

**Methods:**

A total of 30 postmenopausal female patients with mild to moderate stress urinary incontinence, recruited from the Affiliated Hospital of Hebei University from September to November 2019, were selected as the study subjects and treated with lattice carbon dioxide laser therapy. Treatment was given at intervals of one month. The degree of urinary incontinence, the urinary incontinence questionnaire (ICI-Q-SF) score, and the urinary incontinence quality of life scale (I-QOL)) Score, surgical satisfaction, one hour pad test and postoperative complications before treatment and after each treatment of all patients were respectively recorded and compared.

**Results:**

Compared with those before treatment, the grade of urinary incontinence and ICI-Q-SF scores of these 30 patients after each treatment were lower, and their I-QOL scores were higher. The difference of one hour urine pad test was statistically significant (P<0.05), but the follow-up data of three months after the third treatment was close to that of one month after the first treatment. The satisfaction rate of these 30 patients was 76.67% (23/30). After treatment, only one patient presented vaginal itching discomfort on the first day after surgery and the symptoms disappeared three days later. No obvious complications occurred in the other 29 patients.

**Conclusion:**

The treatment of mild and moderate postmenopausal patients with stress urinary incontinence with lattice carbon dioxide laser can effectively reduce the incidence of incontinence and improve the quality of life.

## INTRODUCTION

Stress urinary incontinence (SUI) is defined as the sudden increase in abdominal pressure and the involuntary leakage of urine from the urethral opening.^1^ It is estimated that the incidence of SUI in European countries is 35%, and that in the United States is about 50%.^2^,^3^ However, the prevalence of SUI among adult females in my country is about 18.9%, with the highest prevalence in the 50-59 years old group, even up to 28.0%, posing a serious medical and economic burden to society.^4^ With the aging of the population, the incidence of SUI increases gradually, seriously affecting the quality of life of middle-aged and elderly women. The decline of pelvic floor support function caused by pelvic floor muscle relaxation or injury, the injury of surrounding tissues supporting urethra, and the pathological changes of urethral sphincter itself are the main causes of SUI. Based on this principle, the pelvic floor muscles of middle-aged and elderly women become more relaxed and damaged as they grow older and pregnant, resulting in the inevitable outcome of SUI. In addition to invasive surgical treatment, pelvic floor muscle training method and pelvic floor electrophysiology therapy are currently used clinically in the treatment of patients with mild SUI. The effectiveness of the treatment lasts for a short period of time and requires repeated treatments, but ends up with unsatisfactory effects. Recently, lattice carbon dioxide laser is a new type of treatment method, which is characterized by short treatment time and quick effect.^5^ In this study, the therapeutic effect of lattice carbon dioxide laser on patients with mild to moderate SUI was mainly explored, and the specific details are reported as follows.

## METHODS

Thirty SUI mild to moderate postmenopausal women recruited by Hebei University Hospital from September to November 2019 were selected as research subjects, aged 48-81 years, with an average age of 60.4 ± 7.8 years old. There were six cases of mild SUI and 24 cases of moderate SUI. All patients underwent routine urine test, ultrasonography of double kidney ureter bladder and residual urine, one hour urine pad test, International Consultation Off Incontinence Questionnaire (ICI-Q-SF) score, and incontinence quality of life scale (I-QOL) score. No urinary tract infection could be seen in 30 patients before surgery, no abnormality was found in ultrasound examination of urinary system, with the residual urine volume of < 30ml. The preoperative one hour urine pad test was 7.76 ± 2.70m1, the preoperative ICI-Q-SF score was 8.37 ± 2.08, and the preoperative I-QOL score was 56.93 ± 6.52. Case selection criteria: patients with a history of vaginal delivery, simple SUI, vagina without injury and bleeding, natural postmenopausal (uterine preservation), etc. Exclusion criteria: Patients with injury or active infection in the treatment area, pelvic organ prolapse, history of genital fistula, severe nervous system diseases with urinary incontinence, neurogenic bladder dysfunction, morbid obesity, insulin-dependent diabetes mellitus (Type-I diabetes), having received other energy-based therapies, taking new drugs for urination, etc. Treatment equipment: the treatment equipment used in this study is Ginas laser manufactured by Lumenis, with an output energy of 10mJ, a hexagonal spot shape, a spot size of 12mm, and a spot density of 5%.

### Ethical approval

The study was approved by the Institutional Ethics Committee of Affiliated Hospital of Hebei University (No.: HDFY-LL-2020-021) on November 1, 2019, and written informed consent was obtained from all participants

### Treatment Procedures

patients were treated with lattice carbon dioxide laser therapy, and the method was as follows: Patients were placed in the lithotomy position. (2) The hand tool was placed into the vagina of the patients, and the treatment was started from the top of the vagina. After each laser irradiation, the hand tool was rotated 60° (12, 2, 4, 6, 8, 10 o’clock directions), and moved 1cm towards the vaginal orifice before laser treatment. (3) The irradiation was stopped about 1cm away from the external orifice of urethra, and the treatment was completed. Patients were treated with lattice carbon dioxide laser therapy once a month, three times as a course of treatment. Patients were informed to refrain from sit-bathing for one week after treatment and from having sex life for two weeks.

### Observation Indicators

The degree of urinary incontinence, ICI-Q-SF score, I-QOL score, 1h urine pad test and postoperative complications before treatment, one month after each treatment, and three months after the third treatment of 30 patients were compared.

The degree of urinary incontinence of patients was divided into four grades: asymptomatic, mild, moderate and severe according to ICI-Q-SF.^6^ No urinary incontinence: the ICI-Q-SF score was 0; (2)Mild urinary incontinence: the ICI-Q-SF score was 1-7; (3)Moderate urinary incontinence: the ICI-Q-SF score was 8-14; (4)Severe urinary incontinence: the ICI-Q-SF score was 15-21.

Postoperative quality of life of the patients was evaluated using I-QOL (centesimal system), and the higher the score, the better the postoperative quality of life. The one hour urine pad test is an international self-made standard hour urine pad weight test,^7^ that is, during the evaluation process, the subjects pre weigh the urine pad they wear prior to the test, ingest 500ml water within the prescribed time, complete a series of standardized activities (such as walking, coughing, climbing stairs, etc.), and finally weigh the urine pad again. For the four grades of one hour urine leakage, mild urinary incontinence: 1h urine leakage ≤ 1 ml (1g); moderate urinary incontinence: 1 ml <1h urine leakage <10ml; severe urinary incontinence: 10ml ≤ 1h urine leakage <50ml; extremely severe urinary incontinence: one hour urine leakage ≥ 50ml.

### Final grading of urinary incontinence:

Grade-I is manifested as involuntary urinary leakage when coughing, sneezing or heavy lifting; Grade-II is characterized by urinary leakage when walking fast, skipping rope or slightly strenuous exercise; Grade-III embodied as urine leakage as the body position changes; Grade-IV is manifested as leakage at rest and nocturnal enuresis.^8^

### Statistical analysis

All the data of this study were statistically analyzed by SPSS 20.0 software, and the measurement data were expressed as mean ± standard deviation (x±s). Comparison between groups was performed by paired t test, the count data was expressed in percentage (%), and χ² test was adopted for intra-group pairing. P<0.05 indicates a statistically significant difference.

## RESULTS

The comparison of all indicators before treatment, one month after each treatment and three months after the third treatment among the 30 patients in this group is shown in Table-I. There were statistically significant differences in ICI-Q-SF scores (P<0.01), and no statistically significant differences in ICI-Q-SF scores between 1 month after the first treatment and 3 months after the third treatment (P>0.05). The differences in I-QOL scores were statistically significant (P<0.05); Moreover, the differences in the urine pad test were statistically significant (P<0.05), while there was no significant difference between the urine pad test 1 month after the first treatment and 3 months after the third treatment (P>0.05). All residual urine after surgery were negative. Postoperative satisfaction survey of the patients showed obvious improvement and a significant decrease in the frequency of urine leakage. The results of ICI-Q-SF score and 1h urine pad test showed that the final effect gradually recovered to the state one month after the first treatment. One month after the first treatment, two out of six patients with Grade-I urinary incontinence had no obvious leakage of urine, and 20 of 24 patients with Grade-II urinary incontinence were reduced to Grade-I urinary incontinence (Table-II), with the effective rate of 73.33% (22/30). Three months after the third treatment, 23 of 30 patients with urinary incontinence were degraded or had no leakage, with an effective rate of 76.67% (23/30). After treatment, there were no complications such as dysuria, frequency of urination, urgency of urination, infection, dyspareunia and pain with intercourse. Only one case had increased vaginal secretion on the first day after surgery, which was not treated, and the symptoms disappeared on the third day after surgery.

**Table I:** Comparison of ICI-Q-SF score, I-QOL score and 1h urine pad test before and after lattice carbon dioxide laser therapy (n=30, x±S).

*Item*	*Before treatment*	*1 month after the first treatment*	*1 month after the second treatment*	*1 month after the third treatment*	*3 months after the third treatment*
ICI-Q-SF score	8.37±2.08	4.77±2.32	3.13±1.91	2.23±1.98	4.33±2.96
I-QOL score	56.93±6.52	91.43±4.25	99.90±3.94	101.77±3.86	95.33±6.07
1h urine pad test	7.76±2.70	4.20±1.93	3.20±1.41	2.59±1.43	3.94±2.29

**Table II:** Grading of female patients with SUI before and after lattice carbon dioxide laser therapy.

*Time*	*Grade (case)*		

	*No leakage of urine*	*Grade-I*	*Grade-II*
Before treatment	0	6	24
1 month after the first treatment	2	24	4
1 month after the second treatment	4	26	0
1 month after the third treatment	10	20	0
3 months after the third treatment	7	18	5

### I-QOL Score

There were statistically significant differences between the two groups before and after treatment (P<0.05). ICI-Q-SF score: The scores between the groups one month after the first treatment and three months after the third treatment were P>0.05 (P=0.22), which was not statistically significant, and the remaining scores between the groups were statistically different. one hour urine pad test: The scores between the groups one month after the first treatment and 3 months after the third treatment were P>0.05 (P=0.26), which was not statistically significant.

## DISCUSSION

The current methods for the treatment of SUI are mainly surgical and non-surgical treatment. For moderate and severe patients, the main surgical methods are TVT, TVT-O (transobturator inside-out tension-free urethral suspension), TOT (outside-in transobturator tape), etc.^9^ TVT, pioneered by Professor UImsten of Sweden, involves placing an artificial mesh belt in the middle of the urethra to provide pelvic floor support to keep urine from leaking when applied with abdominal force. The new TVT-O was developed by Dr. De Leval of Belgium, who modified the original TVT sling procedure to allow a safer transobturator membrane pathway that avoids the retropubic space pathway that may damage the bladder. Perioperative safety is the same as most surgeries, and there are certain hidden dangers. Recurrence of SUI after surgery is not uncommon, about 2% - 23%.^10^ It has been suggested in the literature that the risk factors of SUI have a bearing on age, preoperative urethral sphincter dysfunction, obesity, concomitant pelvic organ prolapse and exacerbation, simultaneous POP repair surgery,^11^ hysterectomy, hormone level and nervous system damage.

For patients with mild to moderate SUI, the current non-surgical new treatments for SUI mainly include electromagnetic therapy, stem cell therapy and laser therapy. Electromagnetic stimulation (MS) is a physical therapy that uses electromagnetic waves to stimulate pelvic floor muscles and nerves. MS is currently mainly implemented by an extracorporeal magnetic inner-vation system (Ex MI), which avoids the trouble of inserting electrodes into the vagina, such as intravaginal electrodes, due to the strong penetration ability of electromagnetic waves. Ex MI therapy may also change the excitability of pelvic floor motor nerve fibers, thus improving the ability of urinary continence.^12^ In 2000, injectable myoblasts were applied for the first time in the treatment of SUI and impaired detrusor contractility.^13^ According to histological analysis, the injectable myoblasts can fuse with the urethral sphincter. Mitterberger M et al.^14^ Confirmed the effectiveness of cell therapy for 119 female SUI patients for the first time. Emerging laser treatments are more and more popular in the medical industry at this stage, and the current laser treatments used for SUI treatment are mainly Er-YAG laser and lattice carbon dioxide laser. Lattice carbon dioxide laser therapy was adopted in this study. Its main principle is that each beaming spot is composed of several small laser beams, which are grid-like. The normal tissues existing between each beam point are heated and ablated under the action of carbon dioxide to increase the temperature controllability, which ultimately leads to vasorelaxation, collagen remodeling and synthesis, neovascularization, and elastin formation.^15^ Furthermore, the vaginal tissue is tightened and the urethral closure pressure is increased, thereby improving stress urinary incontinence, restoring the premenopausal state dominated by lactic acid bacteria, and improving sexual experience,^16^ Er-YAG and lattice carbon dioxide laser have similar effects. However, there is no evidence to compare the efficacy of the two kinds of laser therapy.

The subjects of this study are all postmenopausal patients with SUI. Menopause refers to a period of continuous cessation of menstruation for 12 months, occurring at an average age of 47 - 51 years old.^17^,^18^ It is a common physiological phenomenon, which has a bearing on the decrease of estrogen and progesterone secretion. It is a common physiological phenomenon, which has a bearing on the decrease of estrogen and progesterone secretion. Such a decline occurs when the stock of ovarian follicles is exhausted, and is mainly related to social status, nutrition, lifestyle, weight and genetic factors.^19^ A large data study in China shows that even if age is taken as a risk factor, there is a correlation between SUI and menopause (OR=1.26).^20^ For this reason, the elderly postmenopausal patients with SUI were mainly studied.

### I-QOL score:

the scores between the groups were statistically different (P<0.05), and the I-QOL score increased one month after each treatment, indicating that the quality of life of the patients was improved. However, the score at 3 months after the third treatment was higher than that at one month after the first treatment, and lower than that after the second two treatments. Such a trend indicates that the efficacy becomes less and less obvious with the extension of time, but the effect still exists within a certain period of time and patients can still benefit from the treatment. ICI-Q-SF score: the scores between the groups one month after the first treatment and three months after the third treatment were P>0.05 (P=0.22), which was not statistically significant, and the remaining scores between the groups were statistically different. One hour urine pad test: the scores between the groups one month after the first treatment and 3 months after the third treatment were P>0.05 (P=0.26), which was not statistically significant, indicating that with the extension of time, the curative effect was not obvious, but the patients can still benefit from the treatment. To put it in a nutshell, lattice carbon dioxide laser therapy cannot permanently cure postmenopausal patients with SUI, but it still has a certain effect within a certain period of time, with almost no obvious side effects.

### Limitations of this study

There are still some limitations in this study. First of all, the sample size of this study is not large enough. If the sample size can be further expanded, the conclusion may be more convincing. Secondly, this study used its own before and after comparison to illustrate the effectiveness of lattice carbon dioxide laser therapy. In the future, it is best to divide patients into groups and apply lattice carbon dioxide laser therapy and Er-YAG laser therapy respectively and make a comparison to get a more scientific conclusion. In addition, patients in this study should be followed for a longer period of time to further determine the efficacy of lattice carbon dioxide laser therapy.

## CONCLUSIONS

This study is carried out mainly for postmenopausal patients with SUI, with its main purpose to show that laser treatment is effective for mild and moderate stress incontinence regardless of age and menopause, while the effect may last longer for mild SUI. Vaginal laser therapy is currently insufficient to be proved as a conventional minimally invasive treatment for SUI, but the results of this treatment also indicate that considerable initial results can be generated by laser therapy, with acceptable safety and low economic burden. According to the results, it is of great significance for moderate postmenopausal patients with SUI to receive two times of reinforcement therapy. As time goes by, the decision to receive the fourth treatment has not yet been determined, and longer follow-up studies are needed.

### Authors’ Contributions:

**YRW &** **DS:** Designed this study and prepared this manuscript, and responsible for the accuracy and integrity of the work.

**ZYC &** **WZY:** Collected and analyzed clinical data.

**YQZ:** Significantly revised this manuscript.
